# Human Umbilical Cord Mesenchymal Stem Cells Improve Locomotor Function in Parkinson’s Disease Mouse Model Through Regulating Intestinal Microorganisms

**DOI:** 10.3389/fcell.2021.808905

**Published:** 2022-01-20

**Authors:** Zhengqin Sun, Ping Gu, Hongjun Xu, Wei Zhao, Yongjie Zhou, Luyang Zhou, Zhongxia Zhang, Wenting Wang, Rui Han, Xiqing Chai, Shengjun An

**Affiliations:** ^1^ Department of Neurology, The First Hospital of Hebei Medical University, Shijiazhuang, China; ^2^ Hebei Provincial Engineering Laboratory of Plant Bioreactor Preparation Technology, Shijiazhuang, China; ^3^ Research Center, Hebei University of Chinese Medicine, Shijiazhuang, China; ^4^ College of Integrated Chinese and Western Medicine, Hebei University of Chinese Medicine, Shijiazhuang, China; ^5^ Affiliated Hospital of Hebei University of Engineering, Handan, China

**Keywords:** human umbilical cord mesenchymal stem cells, Parkinson’s disease (PD), gut microbiota, inflammation, neurotransmitter, goblet cells

## Abstract

Parkinson’s disease (PD) is a progressive neurological disorder characterized by loss of neurons that synthesize dopamine, and subsequent impaired movement. Umbilical cord mesenchymal stem cells (UC-MSCs) exerted neuroprotection effects in a rodent model of PD. However, the mechanism underlying UC-MSC-generated neuroprotection was not fully elucidated. In the present study, we found that intranasal administration of UC-MSCs significantly alleviated locomotor deficits and rescued dopaminergic neurons by inhibiting neuroinflammation in a PD mouse model induced by 1-methyl-4-phenyl-1,2,3,6-tetrahydropyridine (MPTP, a toxic agent which selectively destroys nigrostriatal neurons but does not affect dopaminergic neurons elsewhere). Furthermore, UC-MSC treatment altered gut microbiota composition characterized by decreased phylum Proteobacteria, class Gammaproteobacteria, family Enterobacteriaceae, and genus *Escherichia-Shigella*. In addition, the neurotransmitter dopamine in the striatum and 5-hydroxytryptamine in the colon were also modulated by UC-MSCs. Meanwhile, UC-MSCs significantly maintained intestinal goblet cells, which secrete mucus as a mechanical barrier against pathogens. Furthermore, UC-MSCs alleviate the level of TNF-α and IL-6 as well as the conversion of NF-κB expression in the colon, indicating that inflammatory responses were blocked by UC-MSCs. PICRUSt showed that some pathways including bacterial invasion of epithelial cells, fluorobenzoate degradation, and pathogenic *Escherichia coli* infection were significantly reversed by UC-MSCs. These data suggest that the beneficial effects were detected following UC-MSC intranasal transplantation in MPTP-treated mice. There is a possible neuroprotective role of UC-MSCs in MPTP-induced PD mice by cross talk between the brain and gut.

## Introduction

Parkinson’s disease (PD) is a common neurodegenerative disorder which occurs due to the loss of dopaminergic neurons. The global prevalence of PD is predicted to be doubled by the year 2040 (Dorsey and Bloem, 2018), making it a faster growing neurodegenerative disorder than Alzheimer’s disease (Group, 2017). It has been shown that pathological mechanisms of PD include α-synuclein aggregation, mitochondrial dysfunction, oxidative stress, autophagy, and neuroinflammation (Charvin et al., 2018). Recent studies have revealed that dysfunction and alteration intestinal barrier in the microbial composition are related to the etiology of PD. In addition, the common non-motor symptoms of PD patients such as constipation begin years before the onset of motor dysfunction (Visanji et al., 2013). The PD mouse model disrupts the intestinal barrier, suggesting that gut–brain interaction plays an important role in PD pathology (Perez-Pardo et al., 2019). In addition, microbiome normalization can improve impaired motor function in MPTP-induced PD mice (Zhou et al., 2019; Sun et al., 2021). These findings indicate that targeting the gut–brain axis is a promising strategy to treat PD.

Mesenchymal stem cells (MSCs) are an important source for tissue repair due to their multifunctional differentiation, easy sampling, rapid expansion, and low immunogenicity, and are also free from ethical issues. MSCs have been used in clinical trials to treat neuropsychiatric disorders such as autism spectrum disorder and multiple sclerosis (Riordan et al., 2018; Riordan et al., 2019). Evidence shows that MSCs have been used to improve intestinal functions and inflammation in inflammatory bowel disease (Soontararak et al., 2018) and to restore gut microbial dysbiosis in various refractory diseases, such as acute liver injury (Dong et al., 2019), rheumatoid arthritis (Li et al., 2020), type 1 diabetes (Lv et al., 2020), and acute lung injury (Sun et al., 2020). Previous studies had reported that administration of MSCs improves motor function and rescues dopaminergic neurons in PD animal models by reducing oxidative stress (Chi et al., 2019), modulating autophagy (Park et al., 2014), and inhibiting neuroinflammation (Kim et al., 2009). However, the molecular mechanisms and interactions between MSCs and gut microbiota in PD remain unknown. What is more, studies demonstrate similar trends in the microbial composition of PD subjects, while pathogenic Gram-negative bacteria (Proteobacteria, Enterobacteriaceae, and *Escherichia*-*Shigella*) and mucin-degrading Verrucomicrobiaceae are increased (Gorecki et al., 2019). MSC treatment can reduce the proportion of Proteobacteria (Lv et al., 2020) and *Escherichia-Shigella* (Sun et al., 2020). Taken together, we assume that the intestinal microbes of PD were also regulated by MSCs.

MSCs are mainly derived from the bone marrow (BM), adipose (AD), and umbilical cord (UC). Compared with BM and AD, UC-MSC harvesting is non-invasive, and cell proliferation is fastest *in vitro* (Li et al., 2015; Fričová et al., 2020). UC-MSCs are not affected by cell contact inhibition, and they are still in a state of proliferation after confluence (Choudhery et al., 2013). These studies indicate that UC-MSCs may be ideal for PD therapy. However, previous studies had reported that UC-MSC transplantation methods mainly focus on stereotactic and intravenous injection in PD animal models (Wang et al., 2016; Chi et al., 2019). There is still a lack of research on the intranasal instillation of UC-MSCs. The advantage of intranasal delivery is brain-targeting; BM-MSCs can be found in multiple brain regions and last up to 4.5 months by intranasal delivery in the PD animal model (Danielyan et al., 2014). Therefore, the present study will further explore the neuroprotective effect of nasal drip transplantation UC-MSCs on PD model mice.

In the present study, we discovered the neuroprotective effects of UC-MSC administration in PD mice. UC-MSCs inhibited reactive gliosis and neuroinflammation and facilitated motor functional recovery in MPTP-treated mice. The neurotransmitter dopamine (DA) in the striatum (ST) and 5-hydroxytryptamine (5-HT) in the colon were also modulated by UC-MSCs. In the same animal, we found that UC-MSCs corrected microbial composition, maintained colonic goblet cells, suppressed colonic pro-inflammatory response, and the activation of the NF-κB pathway in MPTP-treated mice. Our findings provide insights into the effects of UC-MSCs on the brain–gut axis in the PD mouse model.

## Materials and Methods

### Cell Culture and Phenotype Identification

Fresh umbilical cord samples were obtained from normal spontaneous full-term delivery mothers with written informed consent and reserved in a sterilized phosphate-buffered saline (PBS) solution processed within 3 h. The cord was rinsed three times to remove the residue blood and clots, cut into 3-cm-long pieces, and rinsed again in a petri dish until the solution became clear. After blood vessels were removed, Wharton’s jelly was dissected into pieces approximately 0.3 cm^3^ in size and then transferred into culture vessels, with 10 ml mesenchymal stem cell complete medium (Beijing Yocon Biology Co., Ltd.) at 37°C in a 5% CO_2_ incubator. The medium was replaced with fresh medium every 3 days after the initial plating. The cultured cells were passaged when cell confluency reached 80%.

Cells at passage 3 were seeded into 12-well plates at a density of 1.3 × 10^4^ cells per well and observed for 5 days for proliferation measurement. At passage 3, the cells were harvested for phenotype identification through staining with antibodies against CD34, CD45, HLA-DR, CD73, CD90, and CD105 (Sino Biological, Beijing, China) and analyzed using a flow cytometer (Celula, Sichuan, China). An MSC three-line differentiation kit was purchased from Guangzhou Cyagen Biological Co., Ltd. The adipogenic, osteogenic, and chondrogenic differentiation were carried out in accordance with the product instructions. Oil red O, Alizarin Red, and Alcian Blue staining were used to observe the abilities of adipogenesis, osteogenic, and chondrogenic differentiation. UC-MSCs from passages 2 to 5 were used for the experiments.

### MPTP Injury Mouse Model and UC-MSC Treatment

Sixty male C57BL/6 mice (8 weeks old, body weight 22–25 g) were purchased from Vital River Laboratory Animal Technology Co., Ltd. (Beijing, China). The mice were housed in a specific pathogen-free laboratory under a controlled environment with a temperature of 22 ± 3°C and humidity 60 ± 5% at 12-h light/12-h dark cycle. All mice were given free access to food and tap water. The experimental protocols and animal care were strictly in accordance with the approval of the Animal Care and Management Committee of Hebei Medical University.

The mice were randomly assigned to receive either intraperitoneal injection of MPTP or normal saline. The MPTP-induced PD mouse model was conducted as previously described (Xu et al., 2019a). MPTP (30 mg/kg, M0896; Sigma-Aldrich) was injected intraperitoneally once a day for 5 days to produce an experimental PD model.

**FIGURE 1:**
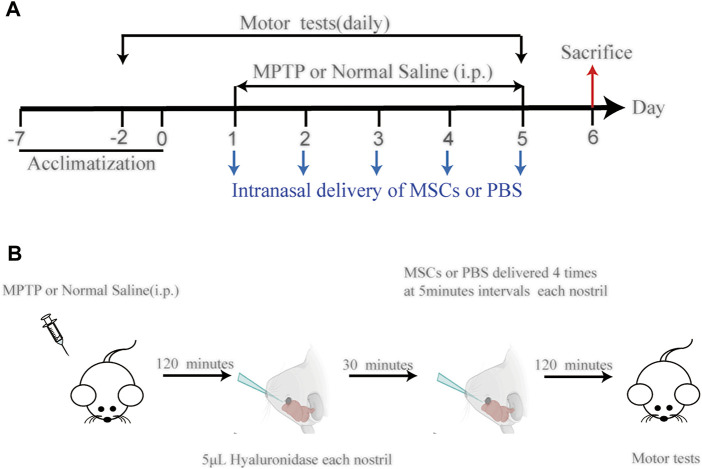
**(A)** Schematic diagram of the experimental design. **(B)** Diagram of the experimental process before sample collection, among which intranasal delivery schema can be found in Rhea EM et al. (2020). DOI:10.3390/pharmaceutics12111120.

The method of UC-MSC administration is modified according to previously published (Long et al., 2017; Narbute et al., 2019; Simon et al., 2019). Intranasal application of UC-MSCs or PBS into MPTP-or vehicle-treated mice was performed 5 days after MPTP injection. Two hours after MPTP injection, each nostril was treated with 5.0 μl of hyaluronidase (100 U; H3506; Sigma-Aldrich) in sterile PBS solution to enhance the permeability of the nasal mucous membrane. Thirty minutes later, 5.0 μl cell suspension was instilled in the nasal cavity with a pipette in a 5-min interval. The daily dose contained 1 × 10^6^ cells/40 μl (Figure 1B).

### Behavioral Test

The motor function was evaluated by two modified pole tests and a traction test (Sun et al., 2018). Behavior training was conducted once a day for three consecutive days, and a behavior test was conducted on the day after the last treatment. The two neurobehavioral tests were performed by investigators blinded to other treatment and group assignment information.

### Immunohistochemistry and Immunofluorescence Staining

Mice were anesthetized with isoflurane, and their brains were gently and quickly removed and post-fixed for 24 h in 4% paraformaldehyde. After being embedded in paraffin, the brains were cut into 3-μm coronal sections by using a microtome (Leica). Sections containing the substantia nigra (SN) were subjected to immunostaining. Briefly, the sections were dewaxed by xylene (I, II, Ⅲ) for 15 min and rehydrated in alcohol (100, 100, 85, and 75%) for 5 min. The tissue sections are placed in citric acid antigen retrieval buffer (pH 6.0) for antigen retrieval. Endogenous peroxidase activity was inhibited by incubation with 3% hydrogen peroxide for 25 min. Then sections were blocked with 3% BSA for 30 min at room temperature and overnight at 4°C with rabbit anti-tyrosine hydroxylase (TH, dilution 1:1000 for immunohistochemistry and 1:2000 for immunofluorescence staining, GB11181, Servicebio). Subsequently, the sections were incubated with horseradish–peroxidase-labeled goat anti-rabbit IgG antibody (dilution 1:200, GB23303; Servicebio) for 50 min at room temperature. The sections then were transferred to fresh 3, 3′-diaminobenzidine for coloration and rinsed with tap water to stop staining. Results were expressed as TH-positive neuron numbers in SN. For immunofluorescence staining, antigen retrieval was performed after the sections were treated with FITC reagent, rabbit-anti-Iba-1 (dilution 1:200, 01919741; Wako), and its corresponding secondary antibody CY3-conjugated goat anti-rabbit IgG (1:300, GB21303; Servicebio) were incubated. For co-expression of TH and GFAP, the mice brain sections were co-incubated with rabbit anti-TH (dilution 1:200, GB11181; Servicebio) and mouse anti-glial fibrillary acid protein (dilution 1:800, GB12096; Servicebio) overnight at 4°C. After being washed in PBS, secondary antibody (488)–conjugated goat anti-rabbit IgG (1:400, GB25303; Servicebio) and CY3-conjugated goat anti-mouse IgG (1:300, GB21301; Servicebio) were incubated. Immunofluorescence images were observed under a fluorescent microscope, and areas of interest were captured and analyzed by ImageJ software.

### Neurotransmitter Measurement by HPLC-MS

The distal colon tissues were collected following previous methods (Li et al., 2019). Striatal DA and colonic DA, 5-HT, 5-hydroxyindoleacetic (5-HIAA) were determined by high-performance liquid chromatography–mass spectrometry (HPLC-MS). The chromatogram collection and integration of each analyte were processed by software Xcalibur 4.0 (Thermo Fisher), and linear regression was performed with weighting coefficients.

### 16S rRNA Sequencing

The fresh feces from mice were collected in sterile tubes and immediately flash-frozen in liquid nitrogen and stored at −80°C until analysis, as previously described (Jiang et al., 2019). The feces samples were transported to OE Biotech Co., Ltd (Shanghai, China) and analyzed on the Illumina MiSeq PE300. After the sequencing data are preprocessed to generate high-quality sequences, Vsearch software is used to classify the sequences into multiple OTUs based on the similarity of the sequences. Then, QIIME software was used to select the representative sequence of each OTU and compare all representative sequences with the Greengenes or Silva database (v. 123) database. Species comparison annotation uses an RDP classifier, and the confidence threshold was 70%.

### Measurement of Cytokines in Serum and Colon

Blood was collected *via* the orbital venous plexus with anticoagulant-free tubes. Blood was centrifuged at 4500 g for 10 min at 15°C, and serum was isolated and stored at −80°C until it was used. The contents of tumor necrosis factor-alpha (TNF-α) and interleukin 6 (IL-6) in serum and the colon were measured using ELISA kits (Proteintech, Wuhan, China) according to the protocol of the manufacturer. The contents of lipopolysaccharides (LPSs) in serum were measured with commercial kits (Nanjing Jiancheng Bioengineering Institute, Nanjing, China) according to the manufacturer’s instructions.

### Periodic Acid–Schiff (PAS) Staining

The colon was fixed in 4% paraformaldehyde, embedded, and cut to 3-μm-thick sections. The sections of the colon were stained with periodic acid–Schiff (PAS) according to a standard procedure. The colonic goblet cells/crypts were analyzed using ImageJ software.

### Western Blot

Colon samples were collected and stored at −80°C. RIPA and PMSF buffer (Solarbio, Beijing, China) was added to extract the protein in the tissue. Protein concentrations were determined using a BCA kit (Solarbio, Beijing, China). Primary antibodies against NF-кB (Cell Signaling Technology, 8242S, 1:1000 dilution), GAPDH (Proteintech, 60004-1-lg, 1:20000 dilution) were incubated at 4°C overnight. The membranes were incubated with horseradish peroxidase (HRP)–conjugated secondary antibodies (Abcam, ab205718, 1:2000 dilution; Proteintech, SA00001-1, 1:2000 dilution) for 1.5 h. The protein bands were visualized by a chemiluminescent substrate (EpiZyme, shanghai, China) and quantitated using ImageJ software.

### Statistical Analyses

Statistical analysis was performed using SPSS 26 software (IBM, United States), and the data were presented as mean ± standard error. Statistical significance between four groups was determined by one-way analysis of variance (ANOVA) with the LSD assay. Bacteria relative abundance differences were performed by Tukey’s honest significant difference (HSD) tests. A *p* < 0.05 was considered statistically significant.

## Results

### Identification of Human Umbilical Cord Mesenchymal Stem Cells (UC-MSCs)

**FIGURE 2:**
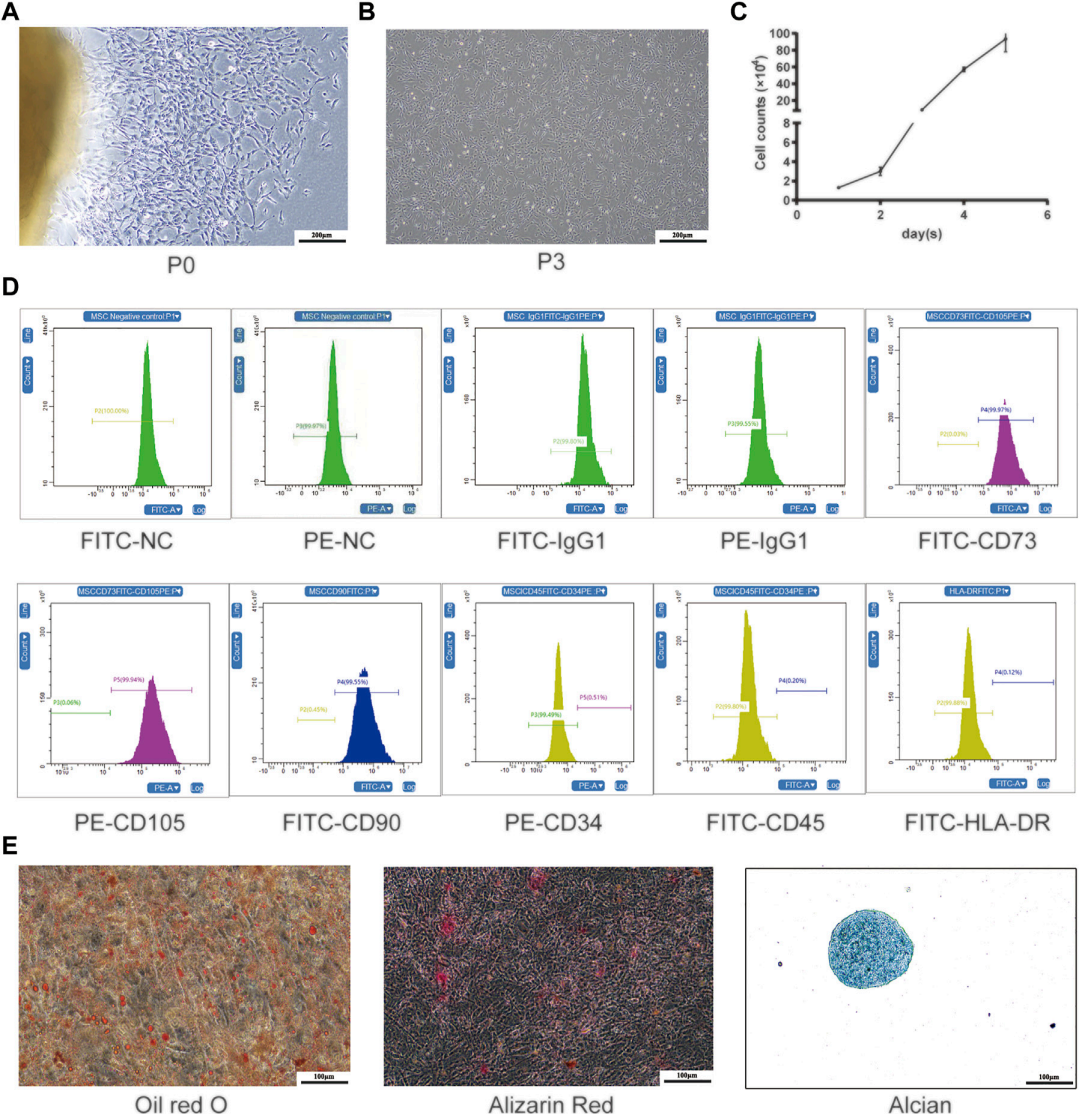
Isolation and production of UC-MSCs. **(A)** Primary cultured UC-MSCs; **(B)** morphological feature and **(C)** growth curve of UC-MSCs at the third passage; **(D)** surface antigen labels of UC-MSCs; **(E)** induced differentiation of UC-MSCs was stained by oil red O, Alizarin Red, and Alcian Blue.

We were able to successfully isolate and culture UC-MSCs from the fresh umbilical cord (Zheng et al., 2020; Yang et al., 2021). The UC-MSCs displayed as spindle-shaped cells crawled out of the tissue pieces when the tissue blocks adhered to the bottom of the culture flask within the medium for 7–10 days (Figure 2A). After culture for 10–14 days, the UC-MSCs were harvested for subculturing. As shown in the third passage, the cell cluster resembled a shoal and small balls in the middle of the cells can be seen in the division phase (Figure 2B). The growth curve indicated that the UC-MSCs grew in an S-shaped curve (Figure 2C), and they continued to proliferate without being influenced by change medium, indicating that UC-MSCs had strong proliferation and self-renewal capabilities. UC-MSCs were identified by harvesting cells at the third passage and analyzed by flow cytometry. These MSCs were positive for CD73, CD105, and CD90 but negative for CD34, CD45, and HLA-DR (Figure 2D). Through induced differentiation of UC-MSCs, massive oil red O-positive lipid droplets and Alizarin Red-stained calcium nodules were formed. Alcian Blue staining shows acid mucopolysaccharides in cartilage tissue (Figure 2E).

### UC-MSCs Improve Motor Function, Restore Dopaminergic Neurons, and Mitigate Microglia-Mediated Neuroinflammation in PD Mice

**FIGURE 3:**
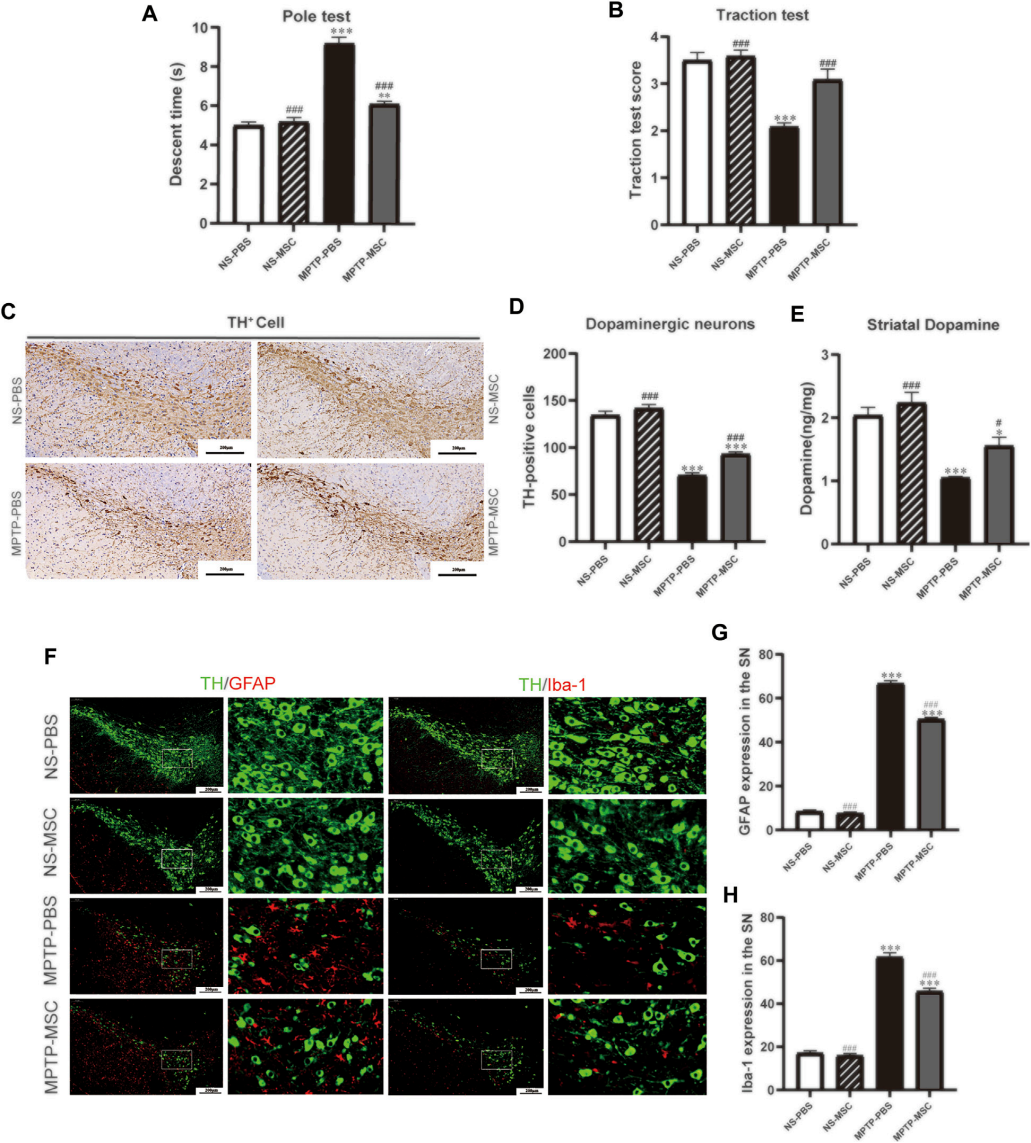
UC-MSCs improved motor function, protected dopaminergic neurons in the substantia nigra and striatum, and alleviated microglia-mediated neuroinflammation in MPTP-induced PD mice. **(A)** Pole test; **(B)** traction test; **(C)** Immunohistostaining for tyrosine hydroxylase (TH) in the SN; **(D)** quantitative analysis of the number of TH-positive cells in the SN; **(E)** content of dopamine was measured by HPLC-MS in the ST. Data of **(A**,**B)** (*n* = 12 per group) are expressed as mean ± SE. Data of **(C**,**D)** (*n* = 3–4 per group) are expressed as mean ± SE. Scale bar: 100 μm (SN). **(F)** Double immunofluorescence staining for TH (green), GFAP (red), and Iba-1 (red) in the SN; **(G)** Quantitative analysis of the number of GFAP positive cells in each group; **(H)** Quantitative analysis of the number of microglia in each group; Data of **(F**–**H)** (*n* = 4 per group) are expressed as mean ± SE. Scale bar: 100 μm (SN). *p < 0.05, **p < 0.01, ***p < 0.001 compared with NS-PBS group, ^##^ *p* < 0.01, ^###^ *p* < 0.001 compared with MPTP-PBS group by one-way ANOVA.

To assess the effects of UC-MSCs on motor function in MPTP-induced PD mice, we used the pole test to determine bradykinesia by measuring the total descent time (Figure 3A) and the traction test to evaluate the muscle strength and equilibrium by measuring the traction scores (Figure 3B). Compared with saline-treated mice, MPTP-treated mice exhibited prolonged pole descent time (9.19 ± 0.32) and lower scores (2.08 ± 0.08) in the traction test. MPTP-treated mice that received UC-MSC treatment had shortened pole descent time (6.11 ± 0.16) in the pole test and increased traction test scores (3.08 ± 0.23). Intriguingly, there was no obvious difference in motor performance between UC-MSC-treated and PBS-treated mice. Therefore, UC-MSC treatment appears to selectively prevent motor dysfunction in PD mice.

To determine the effects of UC-MSCs on the survival of dopaminergic neurons in the SN and DA levels in the ST, we characterized TH expression by immunohistochemistry staining in the SN and HPLC-MS detection in the ST. Immunohistochemistry staining in the tyrosine hydroxylase revealed a significant loss of TH-positive cells in MPTP-PBS mice compared with NS-PBS mice (70.58 ± 2.56 vs 134.46 ± 4.28, *p* < 0.001). MPTP-treated mice received UC-MSCs displayed more TH-positive cells than MPTP-mice received PBS in the SN (70.58 ± 2.56 vs 93.08 ± 2.41, *p* < 0.001). TH-positive cells in the SN did not differ between PBS-treated mice and UC-MSC-treated mice (134.46 ± 4.28 vs 141.71 ± 4.02, *p* < 0.001, Figures 3C,D). HPLC-MS detection showed that the DA levels in the ST dramatically decreased in MPTP-PBS mice compared with NS-PBS mice (1.05 ± 0.02 vs 2.04 ± 0.12, *p* < 0.001). MPTP-treated mice received UC-MSC treatment displayed higher DA levels than MPTP-treated mice thhat received PBS (1.56 ± 0.14 vs 1.05 ± 0.02, Figure 3E). These data confirmed the loss of dopaminergic neurons in the SN and decreased DA levels in the ST induced by MPTP and rescued by UC-MSCs.

To explore the effects of UC-MSCs on microglial phenotype, we analyzed microglia marker Iba-1 and GFAP by immunofluorescence. Double immunofluorescence staining for TH (dopaminergic neuron marker) and GFAP (astrocyte marker) revealed the presence of a higher number of astrocytes in MPTP-PBS mice than NS-PBS mice in the SN (66.67 ± 1.33 vs 8.33 ± 0.80, *p* < 0.001). UC-MSCs significantly decreased the number of astrocytes around dopaminergic neurons compared with MPTP-PBS mice (50.44 ± 0.84 vs 66.67 ± 1.33, *p* < 0.001, Figures 3F,G). Similarly, co-expression of TH with Iba-1 (microglia marker) showed that the microglia number in the SN increased in MPTP-PBS mice compared with NS-PBS mice (61.56 ± 2.08 vs 17.11 ± 1.05, *p* < 0.001), and in MPTP-MSC mice, the microglia number decreased by compared with MPTP-PBS mice (45.67 ± 1.47 vs 61.56 ± 2.08, *p* < 0.001, Figures 3F,H).

### UC-MSCs Modulate Gut Microbiota in PD Mice

**FIGURE 4:**
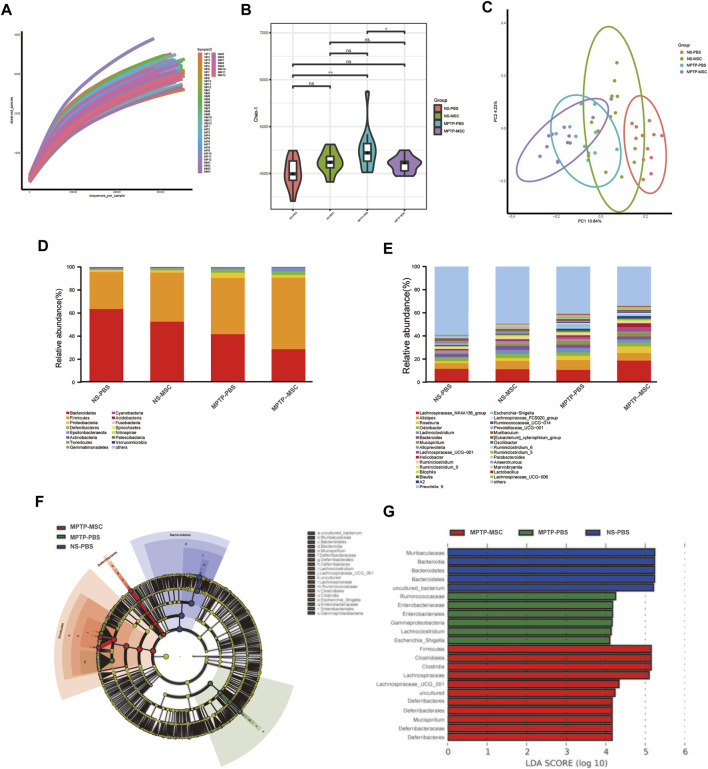
UC-MSCs modulated the composition of gut microbiota in MPTP-induced PD mice. **(A)** Rarefaction curves of samples; **(B)** alpha diversity presented by Chao-1 index; **(C)** PCoA analysis of samples; **(D)** histogram based on relative abundance at phylum level; **(E)** average relative abundance of the microbial community for each group at the genus level. **(F**,**G)** Comparison of gut microbiota using LES among the NS-PBS, NS-MSC, MPTP-PBS, and MPTP-MSC groups at the OUT level. Cladogram **(F)** and histogram **(G)** of bacterial taxa that significantly differed among the three groups (LDA >4.0 and *p* < 0.05). Blue, green, red, or shading in the cladogram depicts bacterial taxa that were significantly higher in the NS-PBS, MPTP-PBS, or MPTP-MSC groups, *n* = 12 per group. *p < 0.05, **p < 0.01 compared with the MPTP-PBS group.

To identify the intestinal microbe phenotypes in responding to UC-MSC treatment, we analyzed the species complexity and difference of bacterial community between groups based on the OTUs and species annotation results. A flat curve was observed as the sequencing quantity increased based on the rarefaction curve, indicating that the sequencing was sufficient for data analysis (Figure 4A). The Chao-1 index, which illustrates the alpha diversity, was closer to the normal level in the MPTP-MSC group compared with the MPTP-PBS group (Figure 4B). The PCoA revealed distinct microbiota composition clustering among NS-PBS, NS-MSC, MPTP-PBS, and MPTP-MSC groups (*p* < 0.001), indicating that MPTP altered the gut microbiota, and UC-MSC administration influences the microbiota composition significantly (Figure 4C). As shown in the histogram at the phylum level, proteobacteria in the MPTP-PBS group were more abundant than the other three groups (Figure 4D). Differential abundance analyses at the genus level revealed that the relative abundance of *Escherichia-Shigella* was significantly increased in the MPTP-PBS group, and the trend was significantly reversed by UC-MSCs treatment (Figure 4E). Furthermore, LEfSe analyses were performed to identify the bacterial taxa that significantly differed after UC-MSC treatment. A significant shift in the microbiota based on relative abundance is shown in the cladogram (Figure 4F). These LEfSe comparisons identified 20 taxa (three phyla, four class, four order, five families, four genera) that were differentially abundant among the three groups (Figure 4F). Significant enrichments in class Gammaproteobacteria, order Enterobacteriales, families Ruminococcaceae and Enterobacteriaceae, and genera *Lachnoclostridium* and *Escherichia-Shigella* were identified in MPTP-PBS mice, while the phylum Bacteroidetes, class Bacteroidia, order Bacteroidales, and families Muribaculaceae were significantly more abundant in fecal samples from NS-PBS mice. Phylum Firmicutes and Deferribacteres, class Clostridia and Deferribacteres, order Clostridiales and Deferribacterales, families Lachnospiraceae and Deferribacteraceae, and genera Mucispirillum and Lachnospiraceae_UCG_001 were significantly enriched following UC-MSC treatment (Figure 4G).

### UC-MSC Treatment Affects the Abundance of Certain Bacteria in MPTP-Induced PD Mice

Further analysis was performed to compare the relative abundance of certain bacteria in these four groups. At the phylum and class levels, MPTP-PBS significantly increased the relative abundance of Proteobacteria and Gammaproteobacteria compared with the NS-PBS group, and UC-MSC treatment decreased the relative abundance of Proteobacteria and Gammaproteobacteria in MPTP-PBS mice. At the order and family levels, the relative abundance of Enterobacteriales, Lactobacillales, Enterobacteriaceae, and Lactobacillaceae was significantly increased in the MPTP-PBS group compared with the NS-PBS group. UC-MSC treatment decreased the relative abundance of Enterobacteriales and Enterobacteriaceae while did not change significantly in the relative abundance of Lactobacillales and Lactobacillaceae in MPTP-PBS mice. At the genus level, the relative abundance of *Escherichia-Shigella*, Alistipes, Lachnoclostridium, and Prevotella 9 significantly increased in the MPTP-PBS group compared with the NS-PBS group. UC-MSC treatment decreased the relative abundance of *Escherichia-Shigella* and Prevotella 9, while did not alter the relative abundance of Alistipes and Lachnoclostridium in MPTP-PBS mice (Table 1).

**TABLE 1:** Top 10 bacteria at differential levels in the four groups.

Relative abundance (%)	Group				*p*-value	
	NP	NM	MP	MM	NP vs MP	MP vs MM
p_proteobacteria	1.91 ± 0.20	2.08 ± 0.24	4.51 ± 1.20	2.40 ± 0.43	0.007	0.027
c_Gammaproteobacteria	0.69 ± 0.14	0.69 ± 0.22	3.29 ± 1.15	0.95 ± 0.39	0.005	0.011
o_Enterobacteriales	0.19 ± 0.05	0.27 ± 0.19	2.87 ± 1.14	0.64 ± 0.35	0.003	0.013
o_Lactobacillales	0.46 ± 0.49	0.38 ± 0.35	0.76 ± 0.57	0.71 ± 0.12	0.005	0.654
f_Enterobacteriaceae	0.19 ± 0.05	0.27 ± 0.19	2.87 ± 1.14	0.64 ± 0.35	0.003	0.013
f_Lactobacillaceae	0.37 ± 0.03	0.35 ± 0.03	0.66 ± 0.04	0.63 ± 0.10	0.001	0.689
g_*Escherichia-Shigella*	0.10 ± 0.03	0.12 ± 0.07	2.42 ± 1.07	0.39 ± 0.17	0.004	0.012
g_Alistipes	4.85 ± 0.68	7.05 ± 0.86	8.55 ± 0.81	6.48 ± 0.89	0.002	0.080
g_Lachnoclostridum	1.15 ± 0.12	3.36 ± 0.42	3.73 ± 0.61	2.83 ± 0.42	0.000	0.146
g_Prevotella_9	0.64 ± 0.03	0.41 ± 0.06	1.64 ± 0.11	1.06 ± 0.06	0.000	0.000

Significant changes of bacteria relative abundance in the four groups. “NP” represents the NS-PBS group, “NM” represents the NS-MSC group, “MP” represents the MPTP-PBS group, and “MM” represents the MPTP-MSC group. Statistical comparison by one-way ANOVA with post hoc comparisons of LSD. Data represent the mean ± SE, *n* = 12.

### UC-MSCs Modulate the Function of Gut Microbiota in MPTP-Induced PD Mice

**FIGURE 5:**
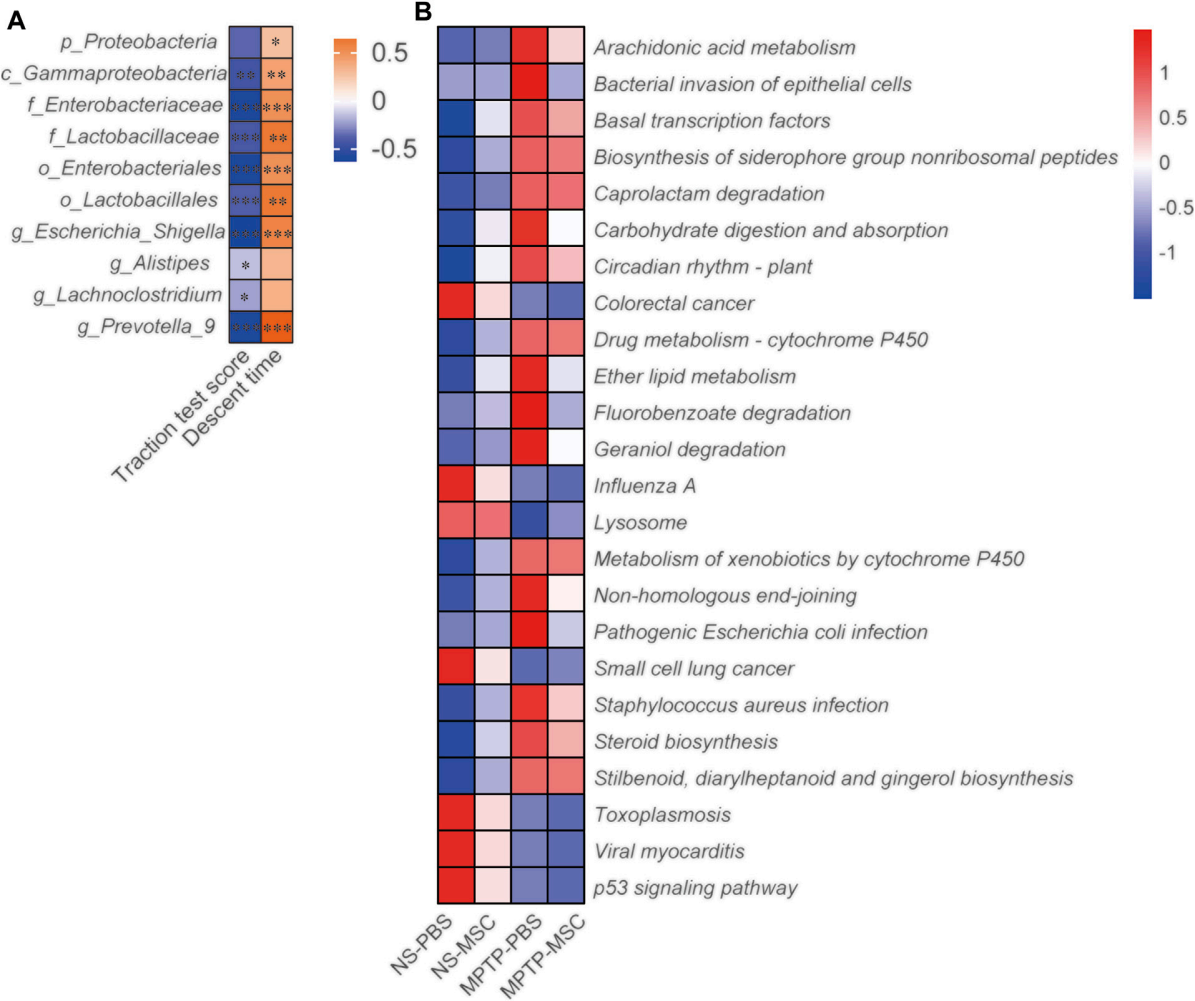
UC-MSCs regulated the effects of gut microbiota in MPTP-induced PD mice. **(A)** The motor function score and relative abundance of gut microbiota were analyzed by Spearman correlation analyses. The association coefficient was performed on the legend of the heatmap. *, **, *** represent the correlation significant (*p* < 0.05, *p* < 0.01, *p* < 0.001, respectively); **(B)** PICRUSt analyses between the four groups. *n* = 12.

We analyzed the correlation between the neurobehavioral parameters and relative abundance of gut microbiota by Spearman’s correlation. Gammaproteobacteria, Enterobacteriaceae, Lactobacillaceae, Enterobacteriales, Lactobacillales, *Escherichia-Shigella*, Alistipes, Lachnoclostridium, and Prevotella 9 were negatively associated with the traction test scores. Proteobacteria, Gammaproteobacteria, Enterobacteriaceae, Lactobacillaceae, Enterobacterales, Lactobacillales, *Escherichia-Shigella*, and Prevotella_9 were positively associated with descent time (Figure 5A). PIRCUSt analysis indicated that the MPTP-PBS group had lower heatmap scores in colorectal cancer, influenza A, lysosome, small-cell lung cancer, toxoplasmosis, viral myocarditis, and p53 signaling pathway and higher scores in arachidonic acid metabolism, bacterial invasion of epithelial cells, basal transcription factors, biosynthesis of siderophore group non-ribosomal peptides, caprolactam degradation, carbohydrate digestion and absorption, circadian rhythm plant, drug metabolism-cytochrome P450, ether lipid metabolism, fluorobenzoate degradation, geraniol degradation, metabolism of xenobiotics by cytochrome P450, non-homologous end-joining, pathogenic *Escherichia coli* infection, *Staphylococcus aureus* infection, steroid biosynthesis, stilbenoid, diarylheptanoid, and gingerol biosynthesis compared with mice in the NS-PBS group. UC-MSC treatment altered the bacterial invasion of epithelial cells, fluorobenzoate degradation, and pathogenic *Escherichia coli* infection compared with the MPTP-PBS group (Figure 5B).

### Effects of UC-MSCs on Pro-Inflammatory Cytokines in Serum and Colon

**FIGURE 6:**
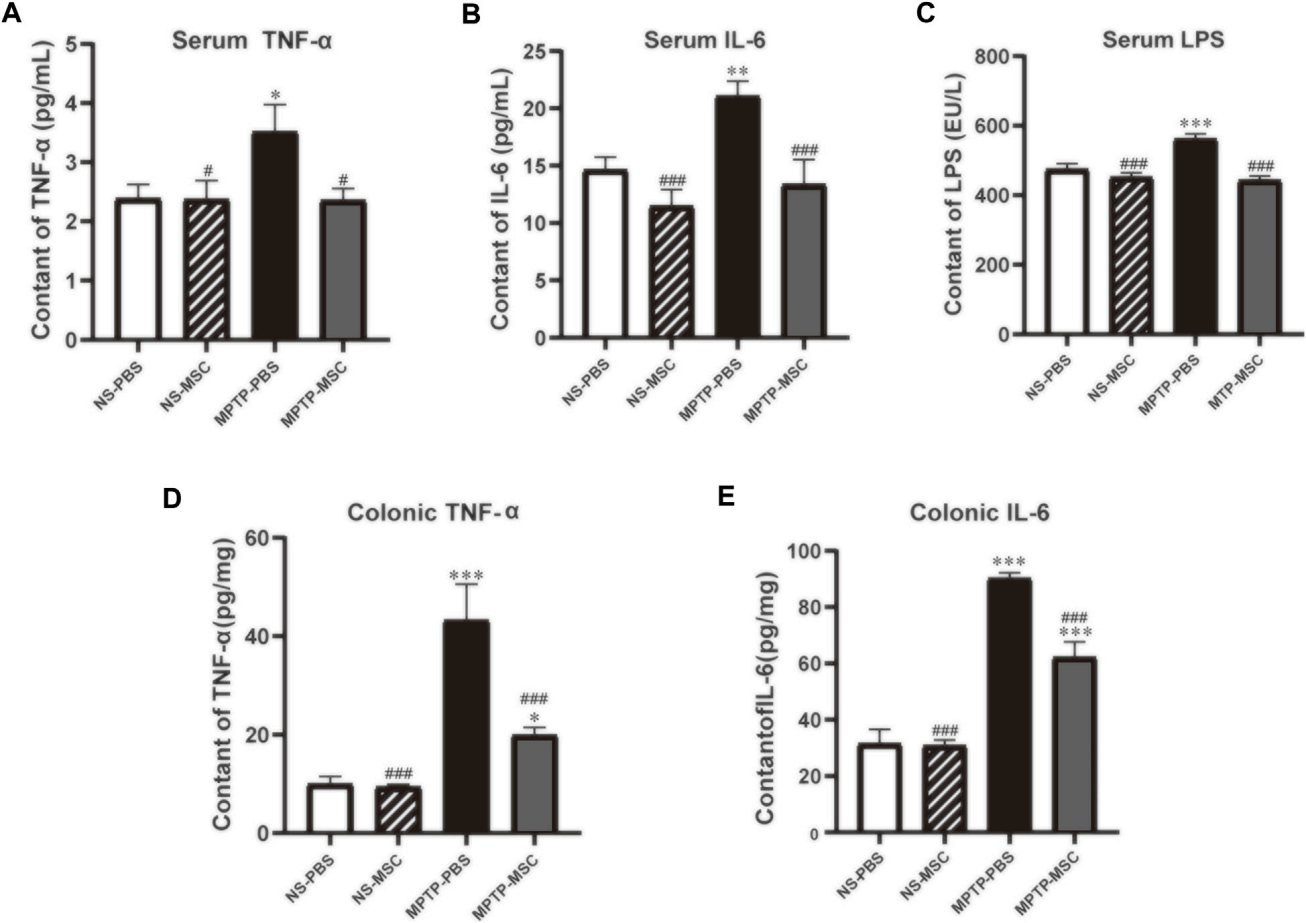
UC-MSCs alleviated serum and colonic inflammatory cytokines in PD mice. Serum levels (pg/ml) of TNF-α **(A)**, IL-6 **(B)**, and LPS **(C)** in mice; colonic levels (pg/mg) of TNF-α **(D)** and IL-6 **(E)** in mice. Data of **(A**–**E)** (*n* = 5–6 per group) are expressed as mean ± SE. *p < 0.05, **p < 0.01, ***p < 0.001 compared with NS-PBS group, ^#^ *p* < 0.05, ^###^ *p* < 0.001 compared with MPTP-PBS group by one-way ANOVA.

The serum and colon levels of pro-inflammatory cytokines are shown in Figure 6. Serum TNF-α, IL-6, and LPS levels were similar between NS-PBS and NS-MSC groups, but PD model mice had higher serum levels of all pro-inflammatory cytokines. After treatment with UC-MSCs, the status of pro-inflammatory was lower than that of the MPTP-PBS group and similar to that of the NS-PBS group (Figures 6A–C). In addition, the effects of UC-MSCs on MPTP-induced pro-inflammatory cytokines in the colon were next explored. Compared with NS-PBS mice, the level of TNF-α and IL-6 in the colon was upregulated in MPTP-induced mice, while the mice treated with UC-MSCs showed lower levels of TNF-α and IL-6 (Figures 6D,E). Generally, UC-MSCs alleviated the levels of pro-inflammatory cytokines in serum and the colon in PD mice.

### Effects of UC-MSC Transplantation on the Level of Neurotransmitter, the Number of Goblet Cells, and the Expression of NF-кB in Colon

**FIGURE 7:**
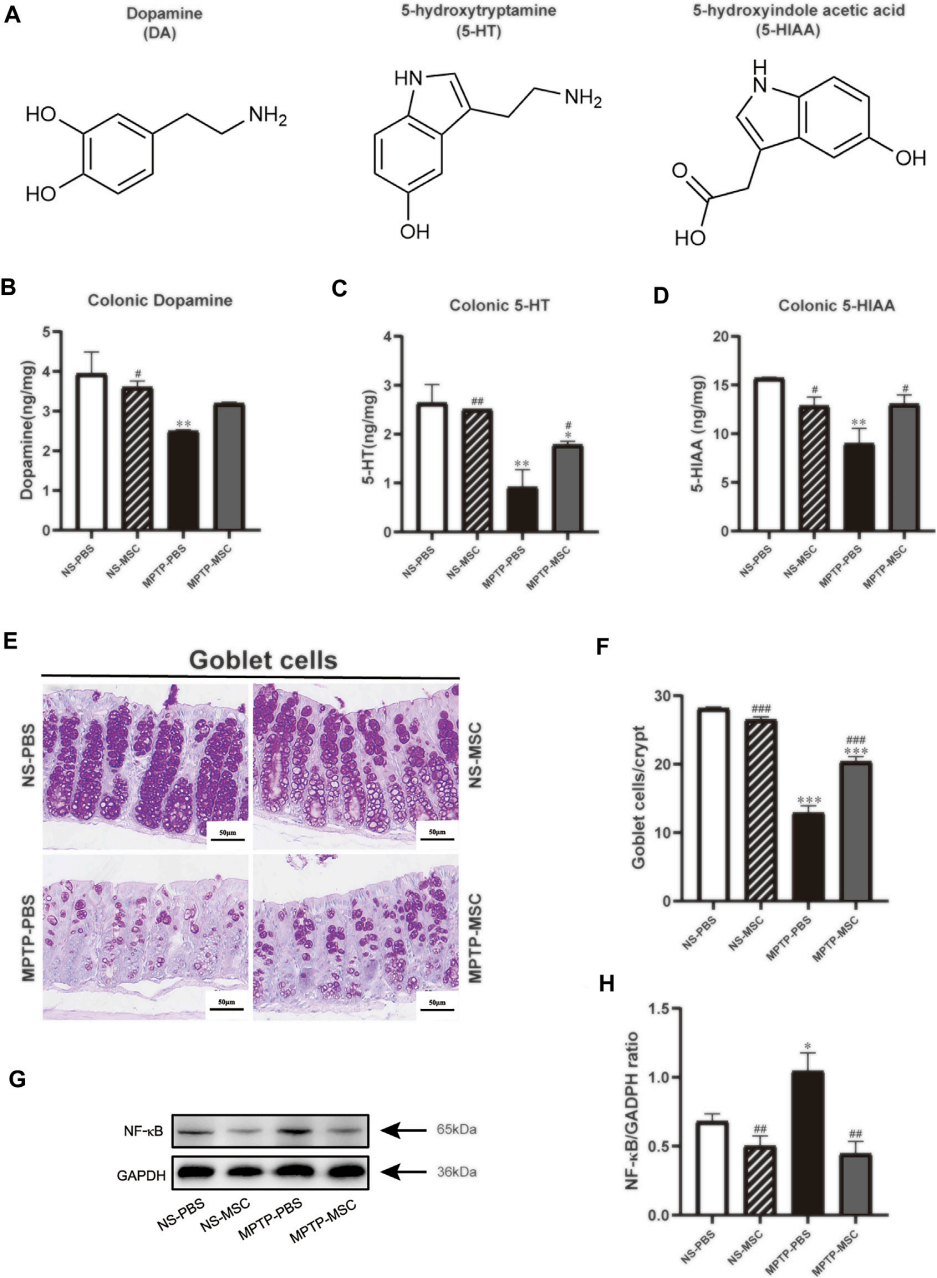
Effects of UC-MSC treatment on the neurotransmitters, goblet cells, and the expression of NF-кB in the colon of MPTP-treated PD mice. **(A)** Chemical structures of the neurotransmitters; **(B**–**D)** DA, 5-HT, and 5-HIAA were analyzed by HPLC; **(E)** PAS staining of the colon (scale bar, 50 μm); **(F)** goblet cells/crypt of colon; **(G**,**H)** Western blot and quantitation of NF-кB protein expression in the colon. Data of **(A**–**H)** (*n* = 3 per group) are expressed as mean ± SE. *p < 0.05, **p < 0.01, ***p < 0.001 compared with the NS-PBS group, ^#^ *p* < 0.05, ^##^ *p* < 0.01, ^###^ *p* < 0.001 compared with the MPTP-PBS group by one-way ANOVA.

We used HPLC-MS to detect the DA, 5-HT, and 5-HIAA in the colon of each group of mice. The content of DA, 5-HT, and 5-HIAA in the MPTP-PBS group was reduced compared to that in the NS-PBS group, and the intervention of UC-MSCs increased the content of 5-HT and 5-HIAA in the colon of PD mice. There is also a growing trend toward the content of DA in the MPTP-MSC group, although it has not reached statistical significance (Figures 7A–D). The goblet cells of the colon are closely related to the function of the intestine. Next, the goblet cells of the colon in every group were detected. As shown in Figures 7E,F, compared with the NS-PBS group, the goblet cells in the MPTP-PBS group were decreased, while UC-MSC treatment significantly attenuated these reductions in MPTP-injury mice, as compared to the MPTP-PBS group. There was no significant difference in the number of colonic goblet cells between the NS-PBS group and the NS-MSC group. Therefore, UC-MSC transplantation can repair the goblet cells in the colon of PD mice. To explore the pathway between the intestinal flora of disbalance and inflammation in the colon, we performed NF-кB by Western blotting. Our results indicated that UC-MSC treatment partially inhibited the expression of NF-кB following MPTP injury (Figures 7G,H).

## Discussion

To our knowledge, this is the first study to determine the effect of UC-MSCs on microbial composition in MPTP-induced PD mouse model. We found that administration of UC-MSCs through intranasal instillation ameliorated motor dysfunction in MPTP-induced PD mice. Furthermore, treatment with UC-MSCs attenuated degeneration of dopamine neurons by inhibiting glial cell activation and decreasing pro-inflammatory cytokine release. In addition, we found that nasal instillation of UC-MSCs changed gut microbiota components, maintained moral mucous barrier, and restrained NF-кB expression. These findings suggest that the brain–gut axis may mediate the beneficial effect of UC-MSCs on motor dysfunction and the protective effect on dopaminergic neurons in PD mice.

A previous study has shown that human umbilical cord blood plasma is beneficial to MPTP-treated rats by reducing pro‐inflammatory cytokines in both the SNpc and intestinal mucosa and dampening inflammation‐associated gut microbiota (Lee et al., 2019). In addition, altering the composition of the gut microbiota ameliorates the neurotoxicity in PD animal models (Dong et al., 2020; Koutzoumis et al., 2020). Our findings in this study provided proof-of-concept evidence that MPTP-treated mice displayed intestinal dysbiosis including impaired goblet cells and subsequently triggered SN neuroinflammation. UC-MSCs administration markedly inhibited the neuroinflammation in the SN and normalized gut microbial dysbiosis, indicating that UC-MSCs play an important role in regulating intestinal disorders in the PD.

In animal studies, intravenous injection of 5 × 10^5^ UC-MSCs to a 6-OHDA–induced PD rat model for 3 days causes significant improvement in motor deficits, and substantia nigra TH^+^ cells significantly increased compared to the vehicle group (*p* < 0.05) (Chi et al., 2019). Another study has shown that in intranasal administration of BM-MSCs to rotenone-induced PD model mice, dopaminergic cellular density in striatum dramatically increased after 10-day transplantation (Salama et al., 2017). However, the therapeutic effect was observed approximately for 5–7 days in the MPTP-induced PD model (Feng et al., 2018; Xu et al., 2019a; Rinaldi et al., 2019). Therefore, the current study explored the effect of intranasal transplantation of UC-MSCs for 5 days on the motor function and dopamine neurons of MPTP-induced PD model mice. We found that intranasal administration of UC-MSCs improved behavioral performance and protected the damaged dopaminergic neurons in the substantia nigra and striatum of PD model mice.

Dysfunction of astrocytic and microglia is involved in the pathogenesis and progression of PD because activated microglia and astrocytes by pathologic α-synuclein (α-Syn) release pro-inflammatory mediators such as TNF-α and IL-1β to promote dopaminergic neuron degeneration (Kam et al., 2020). It has been indicated that MSCs may directly impact glial cells through paracrine (Gharbi et al., 2020), the release of neurotrophic factors (Ko et al., 2018), and macrophage polarization (Lu et al., 2020). Our data showed that intranasal administration of UC-MSCs retained a normal number of astrocyte and microglial cells in the substantia nigra and decreased the level of TNF-α and IL-6 in MPTP-PD mice.

The most salient finding of our study is that UC-MSC administration decreased the relative abundance of *Proteobacteria* in MPTP-induced PD mice. Our results also demonstrated that gut microbial dysbiosis in PD mice is characterized as increases in class Gammaproteobacteria, order Enterobacteriales and Lactobacillales, family Enterobacteriaceae and Lactobacillaceae, and genus *Escherichia_shigella*. It is well known that (Ling et al., 2020) the growth of Gammaproteobacteria, Enterobacteriales, and Enterobacteriaceae of Proteobacteria could trigger the secretion of pro-inflammatory cytokines, which are induced by LPS (Dinh et al., 2015; Shin et al., 2015), and subsequently contribute to the disruption of the intestinal barrier (Litvak et al., 2017). Previous studies have reported that compared with healthy subjects, bacteria in feces from PD patients were higher in Lactobacillaceae, Enterobacteriaceae, and Enterococcaceae, while a reduction in Lachnospiraceae and an increase in Enterobacteriaceae were correlated with motor impairment and disease severity (Pietrucci et al., 2019). Another clinical study has shown that (Xu et al., 2019b) the abundance of Enterobacteriales and Enterobacteriaceae in patients during the first week in the neurological intensive care unit increases the risk of 180-day mortality, whereas a low level of Lachnospiraceae and the enrichment of Lactobacillaceae were associated with postural instability and gait disturbances (Barichella et al., 2019). The facultative anaerobes belonging to the phylum Proteobacteria, such as *Escherichia*, have been reported to be related to colitis (Hu et al., 2020). In addition, we found that UC-MSC treatment decreased the relative abundance of *Escherichia-Shigella*. It has been shown that *Escherichia-Shigella* can secrete amyloid protein to activate microglia (Chen et al., 2020), induce oxidative stress, and release inflammatory factors such as TNF-α, IL-1β, and IL-6 (Harach et al., 2017; Van Gerven et al., 2018). These inflammatory factors may increase the permeability of the intestinal epithelial and blood–brain barrier and subsequently damage the cell in the brain (Lee and Tesh, 2019). Thus, UC-MSC treatment improved gut microbial dysbiosis in the PD mouse model.

The functional pathways involved in the effect of UC-MSCs on the MPTP-induced PD model were assessed by PICRUSt Kyoto Encyclopedia of Genes and Genomes (KEGG) pathway analysis. UC-MSC treatment rescued epithelial cells by preventing bacterial invasion. Interestingly, the number of *Escherichia coli* is increased in inflammatory bowel disease (IBD) patients’ fecal samples as revealed by bacteriological analysis (Mirsepasi-Lauridsen et al., 2019), and MSC therapy enhances *Escherichia coli* clearance in a mice model of bacterial pneumonia (Gupta et al., 2012). Furthermore, a recent study suggested that colonization of Curli-producing *Escherichia coli* accelerates a-Syn pathology in the gut and brain. *Escherichia coli* needs Curli expression to exacerbate a-Syn–induced intestinal and motor disorders (Sampson et al., 2020). Our results showed that UC-MSCs reduced the expression of pathogenic *Escherichia coli* infection, indicating that UC-MSCs play a vital role in intestinal flora modulation. It has been shown that the fluorobenzoate degradation pathway is related to the severity of intestinal inflammation (Montassier et al., 2015). Furthermore, the disappearance of Proteobacteria and the subsequent decreased level of fluorobenzoate degradation improve intestinal *C. difficile* infection (Fujimoto et al., 2021). Strikingly, we found that UC-MSC administration decreased lower heatmap scores involved in fluorobenzoate degradation. This is a promising index to evaluate UC-MSC efficacy. Previous studies have verified the protective effect of geraniol on PD animal models by alleviating a-Syn aggregation, maintaining the mitochondrial function, enhancing antioxidant, and restoring the generation of BDNF and GDNF (Rekha et al., 2013a; Rekha et al., 2013b; Rekha and Inmozhi Sivakamasundari, 2018). Geraniol also targets systemic and local inflammation, dysbiosis, and mucosal damage to alleviate the dextran sulfate sodium (DSS)–induced colitis mouse model. These effects were speculated to be related to Lactobacillaceae (De Fazio et al., 2016). We observed that UC-MSC treatment slightly alleviated geraniol degradation without reaching statistical significance. Thus, future studies are warranted to assess the effect of UC-MSCs on geraniol degradation.

Dopamine and serotonin are major neurotransmitters in the gut in the regulation of nutrient absorption, blood flow, gut microbiome, local immune system, and overall gut motility (Mittal et al., 2017). A decrease in dopamine in mucus in colitis patients is a marker for impaired intestinal mucosal barrier (Magro et al., 2002; Dorofeyev et al., 2013). Furthermore, the level of 5-HT is a key player in regulating mood, sleep, and behavior disorders and is linked to imbalanced 5-HT in the gut (Delgado et al., 1990; Berger et al., 2009). We found that UC-MSC treatment significantly elevated the reduced colonic dopamine, 5-HT, and 5-HIAA levels in MPTP-treated mice. Furthermore, the observed effects of UC-MSCs on colonic neurotransmitters are consistent with the degree of colonic injury. Consistent with a recent study showing that MSCs increase goblets, where the mucus is mainly synthesized, stored, and released in experimental colitis (Alves et al., 2019), we found that UC-MSCs recovered the reduced number of goblets in the MPTP-treated mice. It has been shown that the intestinal microbiota can influence the properties of the colonic mucus layer, and mice with a penetrable mucus layer had higher levels of *Proteobacteria* in the distal colon mucus (Jakobsson et al., 2015). Thus, further investigation is needed to elucidate the precise mechanism through which other bacteria interact with mucus production.

It has been shown that the expression of α-Syn in the brain positively correlated with the degree of α-Syn in the intestinal wall since injection of Lewy bodies into the striatum induces enteric synucleinopathy in baboon monkeys (Stolzenberg et al., 2017). In addition, microbial dysbiosis can lead to increased gut mucosal permeability and inflammation, which in turn trigger α-synuclein aggregation [77]. Previous studies have shown that MSC intervention reduces the expression of α-Syn aggregates through the secretion of metal matrix protease (MMP2) (Oh et al., 2017) and induction of autophagy (Park et al., 2014). UC-MSCs may also reduce the increase in Lewy bodies in the brain and subsequently reduce the abnormal accumulation of α-Syn in the intestine, which further alleviates the inflammation of the gut. Microbial dysbiosis can lead to increased gut mucosal permeability and inflammation, which in turn trigger α-synuclein aggregation (Dalile et al., 2019). Consistently, we found that the level of TNF-α and IL-6 and the expression of NF-кB were decreased in the colon, indicating that UC-MSCs exert anti-inflammatory effects in the colon in MPTP-treated mice.

In summary, we found that UC-MSCs modulated microbial composition in an MPTP-induced PD mouse model. UC-MSCs ameliorate motor dysfunction and repair degeneration of dopamine neurons through inhibiting activated glial cells, decreasing the release of pro-inflammatory cytokines, maintaining the normal mucous barrier, and restraining the expression of NF-кB. Our findings suggest that the brain–gut axis may be a potential mechanism underlying the beneficial effect of UC-MSCs on PD mice.

## Data Availability

The original contributions presented in the study are publicly available. This data can be found here: https://www.ncbi.nlm.nih.gov/sra/PRJNA784361.

## Abbreviations

DA, dopamine; ELISA, enzyme-linked immunosorbent assay; GFAP, glial fibrillary acid protein; 5-HIAA, 5-hydroxyindoleacetic; 5-HT, 5-hydroxytryptamine; Iba-1, ionized calcium-binding adaptor molecule 1; LPS, lipopolysaccharide; MSCs, mesenchymal stem cells; PD, Parkinson’s disease; SN, substantia nigra; ST, striatum.
